# Improved breast cancer risk prediction using chromosomal-scale length variation

**DOI:** 10.1186/s40246-025-00776-z

**Published:** 2025-06-11

**Authors:** Yasaman Fatapour, James P. Brody

**Affiliations:** Department of Biomedical Engineering, University of California, Irvine, 3120 Natural Sciences II, 92697 Irvine, CA USA

## Abstract

**Introduction:**

Early diagnosis of breast cancer leads to higher long-term survival rates. The development of a germline genetic test, or polygenic risk score, to identify women at high risk of breast cancer holds the potential to reduce cancer deaths. However, current tests based on SNPs do not perform much better than predictions based on family history and perform significantly worse in populations with non-European ancestry. We have developed an alternative method to characterize a genome, called chromosomal-scale length variation, which can be applied to polygenic risk scores.

**Objective:**

The objective of this paper is to characterize a breast cancer genetic risk score based on chromosomal-scale length variation using the NIH All of Us dataset in different self-identified racial groups when trained on different populations.

**Methods:**

We used the NIH All of Us dataset to compile a dataset with 4,533 women who have been diagnosed with breast cancer (including 440 who self-identified as Black) and 44,518 women who have not. We acquired, through All of Us, genetic information for each of these women. We computed a set of 88 values for each woman in the dataset, representing the chromosomal-scale length variation parameters. These numbers are average log R ratios for four different segments from each of the 22 autosomes. We used machine learning algorithms to find a model that best differentiates the women with breast cancer from the women without breast cancer based on the set of 88 numbers that characterize each woman’s germline genome.

**Results:**

The best model had an AUC of 0.70 (95% CI, 0.67–0.73) in the All of Us population. Women who scored in the top quintile by this model were nine times more likely to have breast cancer when compared to women who scored in the lowest quintile.

**Conclusion:**

In conclusion, we found that this method of computing genetic risk scores for breast cancer is a substantial improvement over SNP-based polygenic risk scores. In addition, we compared models trained on populations of only White women and only Black women. We found that the models trained only on White women performed better than models trained only on Black women when tested on only White women. We did not see a significant difference between the two models when tested on only Black women.

## Introduction

About 287,000 new breast cancer cases are reported annually in the United States, along with over 43,000 deaths (as of 2022 [1]),. Incidence and mortality rates vary significantly among U.S. subpopulations, with notable differences by race and ethnicity [1]. Over 30% of breast cancer cases are attributable to heritable causes. Identifying these inherited factors might contribute to improvements in screening and prevention strategies [2]. Early diagnosis of breast cancer leads to much higher long-term survival rates [3]. The development of a germline genetic test to identify women at high-risk of breast cancer holds the potential to reduce cancer deaths. This reduction could occur either through surgical removal of tumors before they spread or prophylactic removal of the organs before any cancer is detected.

Currently, prophylactic removal of the breast is practiced in high-risk women who test positive for particular BRCA1/2 variants [4, 5]. While a positive BRCA1/2 test is highly predictive of breast cancer, a negative test does not rule out breast cancer. The prevalence of pathogenic variants in the BRCA1 and BRCA2 genes is estimated to be in approximately 1 in 400 to 1 in 500 individuals in the general population [6]. Notably, only about 5–10% of breast cancers in the U.S. are associated with these specific mutations [7, 8], highlighting a substantial need for effective genetic tests targeting the remaining 90–95% of cases.

The most common approach to addressing this challenge involves using polygenic risk scores [9–12]. These scores are typically computed from linear combinations of genetic variants called single nucleotide polymorphisms (SNPs) that occur more frequently in breast cancer patients than in the general population.

However, structural variations in the genome, particularly copy number variations (CNVs), are increasingly recognized as important contributors to genetic predisposition to cancer. CNVs can disrupt gene function, alter gene dosage, or impact regulatory regions, thereby influencing cancer susceptibility through diverse mechanisms [13–15]. Despite these insights, current polygenic risk scores primarily rely on SNP data and overlook structural genomic alterations [16].

The effectiveness of a classification model or diagnostic test can be measured by the area under the receiver operating characteristic curve (AUC). The best polygenic risk test for breast cancer risk prediction currently achieves an AUC of approximately 0.60 [2, 9], which is only slightly better than random guessing (AUC = 0.50). A perfect test would have an AUC of 1.0.

Among the predictive models used for breast cancer risk assessment, the Gail model, which was originally developed based on data from White women, has an AUC of 0.58 [17, 18]. This statistical model incorporates an individual’s personal information, including parameters related to first-degree relatives diagnosed with breast cancer. Another risk estimate model, the Tyrer-Cuzick model [19], provides a more detailed genetic picture by considering BRCA1/BRCA2 mutations. This model performs better, achieving an AUC of 0.63 [20].

Current polygenic risk scores are not significantly better than the Tyrer-Cuzick model. These polygenic risk scores have known limitations. They rely on linear combinations of variants and do not account for non-linear interactions [10–12, 21–23], such as epistatic effects, where the impact of one genetic variant on breast cancer risk depends on the presence or absence of other variants. While statistical techniques based on non-linear combinations (such as machine learning models) exist, they require a large number of samples (cancer patients) relative to the number of variables (SNPs). Given that millions of SNPs are necessary to characterize a human germline genome, applying these strategies would necessitate an unrealistic dataset of tens of millions of patients.

To address this limitation, we have developed a novel approach for genome characterization, which we call chromosomal-scale length variation (CSLV) [24]. Unlike traditional methods, CSLV captures large-scale structural variation by computing the average copy number variation across chromosome-sized regions. Using this method, we can characterize a genome with a few dozen numbers, substantially reducing dimensionality while preserving critical biological information. Importantly, using CSLV, we can construct highly predictive genetic risk scores from relatively small cohorts, requiring as few as a hundred cancer patients. The effectiveness of polygenic risk scores can vary depending on the racial and ethnic makeup of the training set and test set [25–28]. Because most genetic data have been collected in homogeneous White populations, most current polygenic risk scores perform best in White populations [25]. Another challenge of developing a polygenic risk score in a minority population is collecting enough data from members of that population. Since the methods we have developed using CSLV work with smaller datasets, we can also create genetic risk scores for smaller minority populations, where the number of patients is limited.

The objective of this paper is to develop and evaluate a breast cancer genetic risk score, or prediction test, based on CSLV using the NIH All of Us dataset. In addition, we assess the model’s performance across different self-identified racial groups to characterize the potential bias introduced by the model.

## Methods

To test how well chromosomal-scale length variation can predict breast cancer among different racial/ethnic backgrounds, we acquired and analyzed germline genetic data from a large diverse dataset, All of Us. The All of Us Research Program is a national initiative led by the National Institutes of Health (NIH) aiming to build a diverse health database to advance precision medicine. The All of Us study has enrolled more than 848,000 participants as of April 2025, with 80% of them coming from underrepresented communities [29]. The All of Us workbench provides researchers access to a wide range of data, including electronic health records, Fitbit data, surveys, socioeconomic information, and genetic data (whole genome sequencing and microarray analysis). Access to the All of Us Researcher Workbench requires completion of the Responsible Conduct of Research training and adherence to strict data use agreements to ensure participant privacy.

Genetic data is being released in phases [29]. The first phase, controlled tier 6, provides microarray data with 1,814,517 genetic variants for each of the 165,127 participants. This analysis is based on tier 6 data.

In the All of Us study, each person’s germline DNA was extracted from blood or saliva sample and was analyzed with the Illumina Global Diversity Array. To access, organize, and analyze this dataset we used the dedicated researcher workbench, a cloud-based platform for data analysis and model development. Genetic data on the platform was accessed from the Hail matrix [30]. A Hail matrix is a data structure used in genetic analysis. It provides a scalable and efficient framework for processing large-scale genetic datasets. This data analysis was conducted on the integrated cloud-based Jupyter notebook environment using the Python programming language on the designated workbench.

### Computation of chromosomal-scale length variation

We characterized a person’s genome using a small set of numbers, rather than a large set of SNPs [31]. Specifically, we extracted the log R ratio (LRR) values from the Hail Matrix entry field for each patient. The LRR values were reported for each 1.8 million locations across the chromosomes. LRR indicates the logarithmic ratio of observed signal intensity, measuring the copy number of genetic material at a specific locus. By computing the average LRR values over a chromosome or a segment of it, we obtained a nominal length for each chromosome. We call this set of numbers the chromosomal-scale length variation (CSLV). An LRR value of 0 indicates the presence of two copies at the locus, while higher values indicate duplications and lower values indicate deletions. In this study, we initially quantified the germline DNA of each participant with 22 numbers corresponding to the average log R ratio (LRR) values of all the SNP markers within that chromosome. The analyzed results of all 22 chromosomes from participants in controlled tier 6 were stored in a data table, and this CSLV data table was utilized for machine learning model development.

**Fig. 1:**
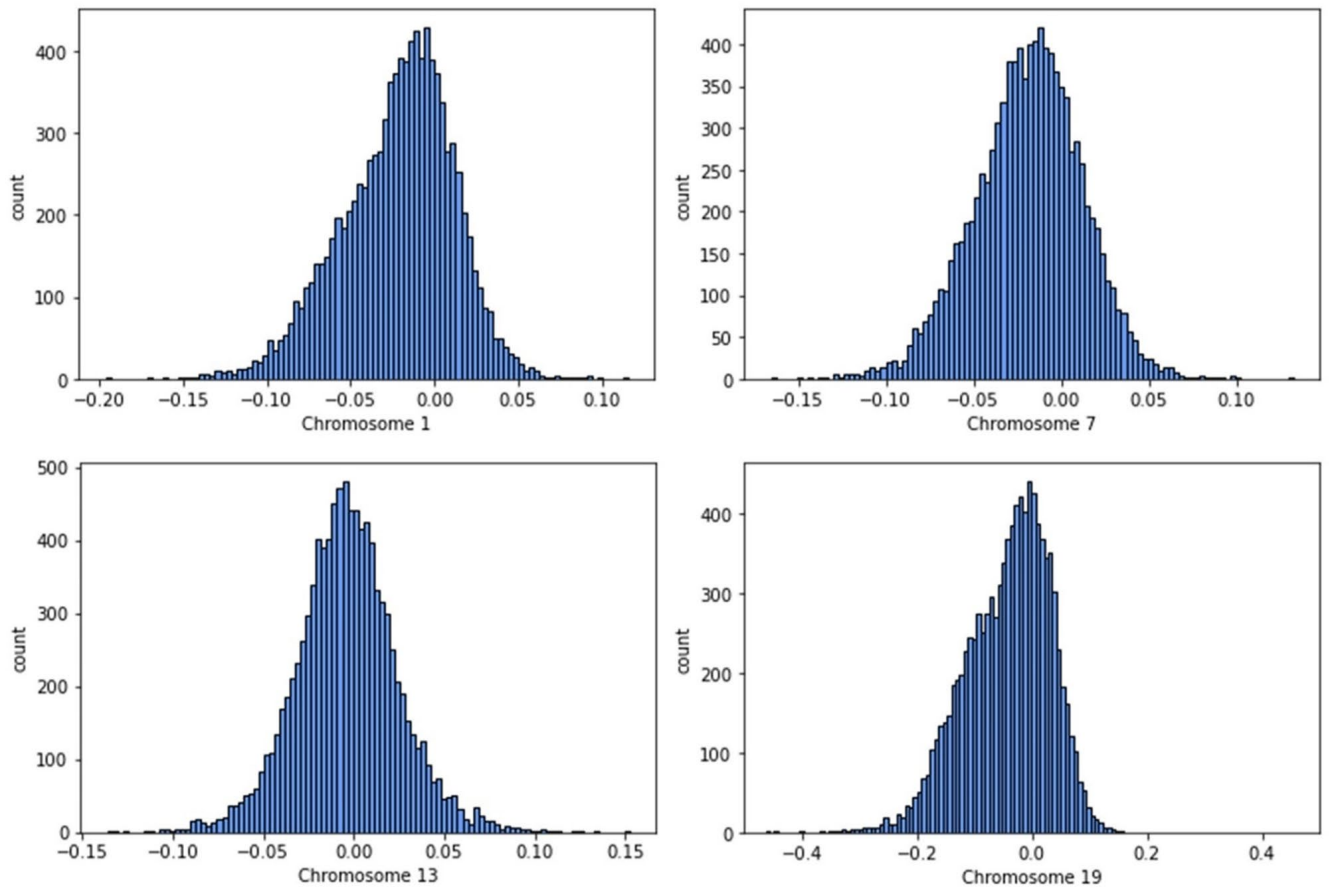
The distribution of relative chromosome lengths obtained from DNA samples in the All of Us dataset for chromosomes 1, 7, 13 and 19. These histograms were plotted for average LRR values of 10,000 participants that were randomly selected from 165,127 participants in the All of Us dataset

Figure 1 presents a histogram that illustrates the distribution of CSLV values, or relative chromosome lengths, obtained from DNA samples in the All of Us dataset, Tier 6, specifically for chromosomes 1, 7, 13, and 19. A value of “0” represents the nominal average chromosome length. This visualization illustrates the extent that this measurement varies across the population, suggesting that this measurement might be appropriate for characterizing a genome.

To improve the resolution of our genome characterization, we further divided each chromosome into four equal-length segments and computed an average LRR value for each segment, resulting in a total of 88 features per individual. This segmentation strategy was chosen to capture more localized structural variations while maintaining a manageable number of parameters. Increasing the number of segments per chromosome beyond four would have significantly increased computational demands, processing time, and memory usage, especially given the size of the All of Us dataset. Therefore, the four-segment approach represented a practical balance between resolution and computational feasibility.

### Machine learning

To train and test machine learning models, we used the H2O AI environment. Specifically, we used H2O’s AutoML function. Given a data frame, where the first column is a binary True/False value indicating whether the row represents a woman with or without breast cancer and the other columns contain the CSLV values, the AutoML function seeks to identify the best way to differentiate between the True and False rows based on the CSLV values. This AutoML function explored various machine learning algorithms and assessed different hyperparameters for each algorithm. The AutoML function evaluated models based on gradient boosting machine (GBM), distributed random forest (DRF), deep learning, logistic regression, generalized linear models (GLM), and ensemble models built from combinations of these five types of models. The AutoML function is provided a time (in seconds), and it uses that time to test several types of models, optimize the hyperparameters for those models, and after the given time, provides the best model, along with a scoreboard of how well other models performed.

We conducted a case/control study to distinguish women with breast cancer cases from those women without any form of cancer. Using the cohort and dataset builder tools within the NIH All of Us Researcher Workbench, participants for each class were identified. From these two separate classes (those women with a malignant breast tumor and a control group consisting of women who have never had any type of cancer), we selected participants with available genetic information and created two distinct case-control tables. These tables only included those patients who had germline DNA microarray measurements within the dataset. The demographic characteristics of this dataset are presented in Table 1.

**Table 1:** This table quantifies the distribution of women in the dataset by self-identified race in the positive and negative classes for female breast cancer. The positive class includes women with a diagnosis of malignant breast tumor, while the control group comprises cancer-free women

Class	Race Distribution					Sex	Total
	White	Black	Asian/Middle Eastern	Other	Unknown	Female	
Positive	3342	440	129	64	558	4533	4533
Negative	17109	13436	1796	1318	13235	44518	44518

The dataset included approximately ten times as many women in the negative class, compared to the positive class. Due to the unbalanced nature of the data, undersampling of the negative class was performed. Specifically, negative class participants were randomly selected and age-matched with positive class participants, while maintaining a 40:60 ratio between the positive and negative classes. This ratio was selected to mitigate the bias-variance trade-off commonly encountered in highly imbalanced datasets [32]. By slightly preserving the natural class imbalance, we aimed to improve model generalization and maintain clinical relevance, while still ensuring that the positive class was adequately represented during model training. Once the finalized dataset was constructed, 20% were set aside as a test set, while the other 80% were used for the AutoML training/testing/validation. The AutoML function tries different machine learning algorithms to develop an accurate polygenic risk score. We used H2O’s AutoML in conjunction with Python within the Researcher Workbench to train and test various algorithms. Each algorithm was set up with a maximum run time of 900 s, and 5-fold cross-validation was employed on the training dataset to train the models. During each run, the cross-validation predictions were retained on the leaderboard. Additionally, the trained model’s performance was evaluated on the unseen test split of the data (20% dataset). To support reproducibility, the analysis code used in this study is available upon request in accordance with All of Us Researcher Workbench policies.

## Results

**Fig. 2:**
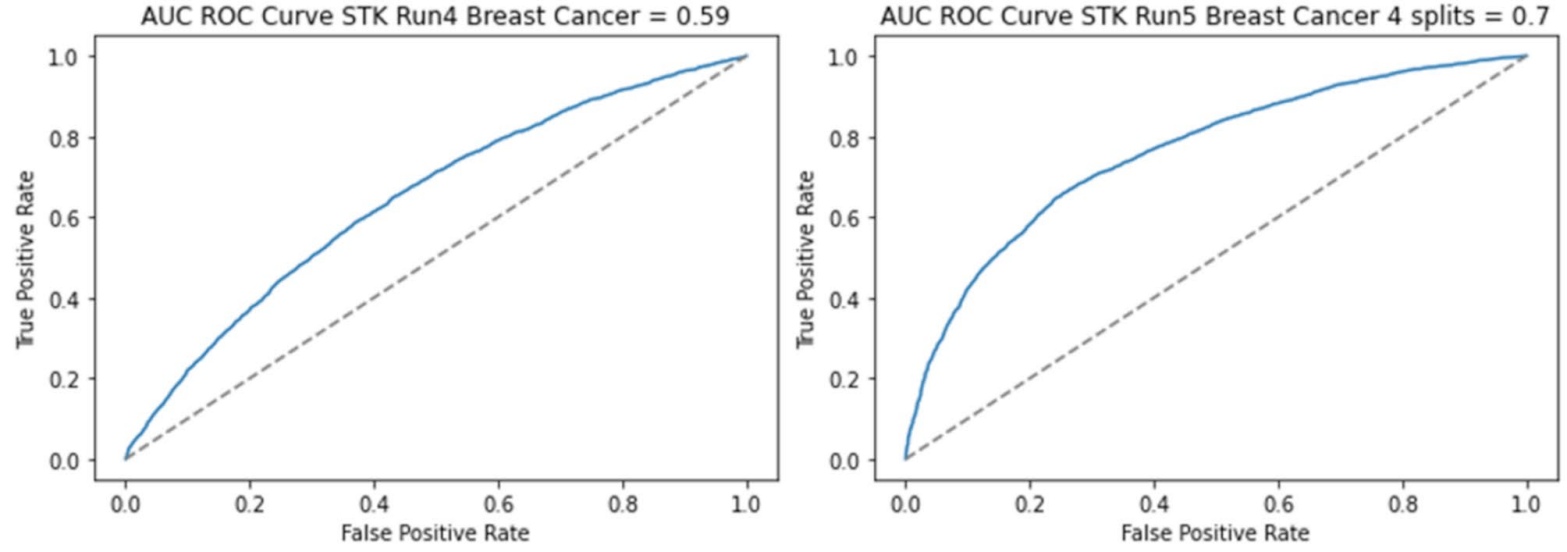
This figure presents the receiver operating characteristic curves for two different models. The curve on the left is based on a model that represented each person with 22 chromosomal-scale length variation parameters, one for each of the 22 autosomes. This model had an AUC of 0.59. The curve on the right is based on a model that represented each person with 88 parameters, four for each of the 22 autosomes. It had an AUC of 0.70. This result indicates that the AUC increases (the predictions are more accurate) when more detailed information is included

For the first classification problem, which involves assessing the risk of malignant breast tumors, we analyzed data from 4,533 women with breast cancer and 44,518 women without breast cancer. Initially, we utilized 22 numbers to characterize each person’s genome, each of the 22 numbers represented the average of all the log R ratio (LRR) values reported for each SNP marker within each chromosome. We did not use data from the X or Y chromosomes. Our classifier achieved an average AUC of 0.60 (SD = 0.015) for identifying breast cancers, as illustrated in Fig. 2a.

These results indicate that this approach is just as effective in predicting breast cancer status as other available polygenic risk scores. To improve the model’s performance, we split each of the chromosomes into four parts and computed a chromosomal-scale length variation number for each quarter of a chromosome (88 numbers). This approach enables us to capture more detailed information regarding the structural variability of the genome. By utilizing these 88 numbers instead of the initial 22 numbers, a more comprehensive machine learning model was developed that incorporates a broader range of genetic information. The evaluation criteria and model’s characteristics remain consistent with the previous approach.

**Fig. 3:**
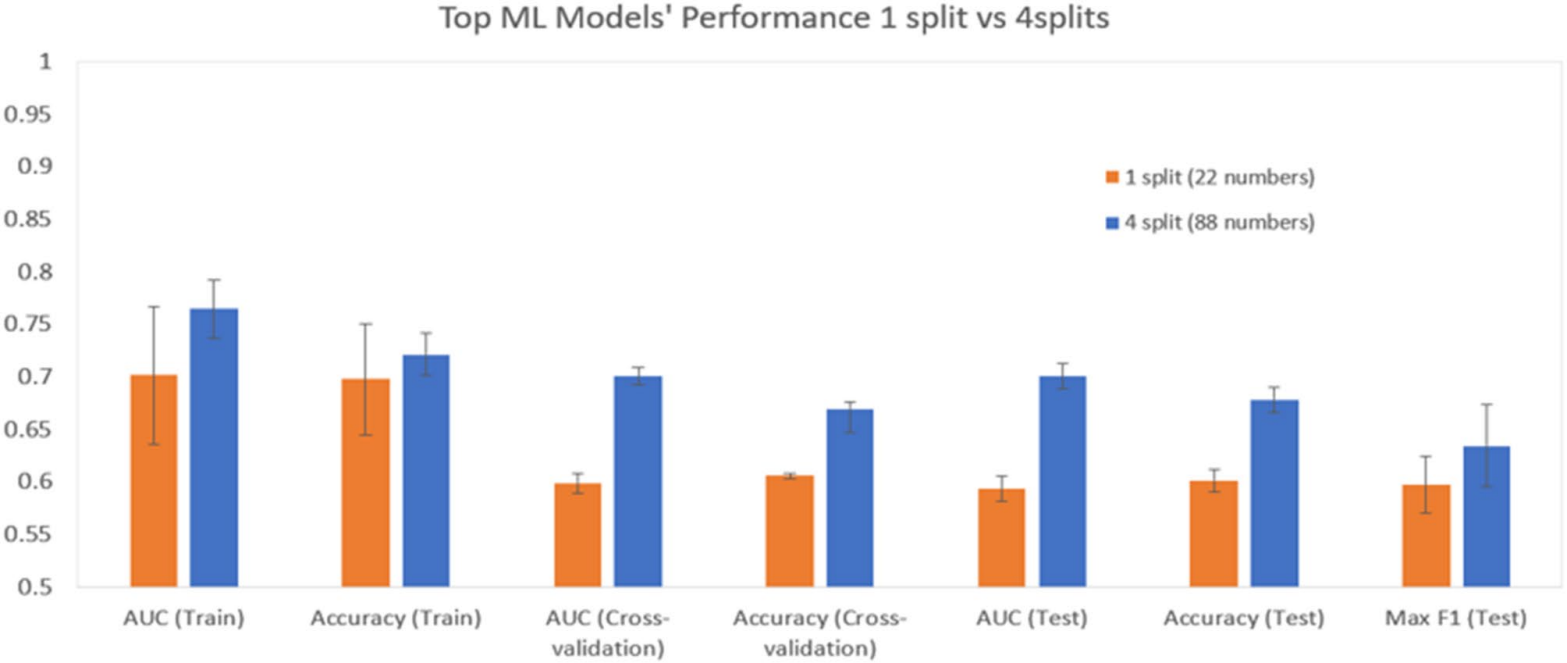
This figure compares the performance of the two different models, using either 22 numbers (one per autosome) or 88 numbers (four per autosome). The performance was evaluated by calculating various metrics on the training, cross validation, and test sets. For all these runs the best classifier was a stacked ensemble which is the optimal combination of a collection of prediction algorithms

Using the same classes in Table 1 with 88 CSLV measurements instead of 22, we conducted multiple runs with consistent hyperparameters, timeframes, and workspace configurations and achieved an average AUC of 0.70 with standard deviation of 0.01 on the test data, see Fig. 2b. This represents a significant increase in predictive accuracy, see Fig. 3.

We next tried to simulate a real-world application. We used a trained model, and a previously unseen test set to predict whether a woman in that test set had breast cancer or not. The model returns a score for each of the 2,015 women in the test set. We assessed the accuracy of the model by ranking each woman based on the assigned score and evaluating the model’s performance across quintiles. A higher score indicates the model believes the woman is more likely to have breast cancer. We calculated the odds ratio of the model within each quintile. Table 2 displays the odds ratios calculated for each respective quintile relative to the entire test population, indicating the increased likelihood of developing breast cancer associated with higher scores. For instance, in our findings, the top 20% of women (ranked according to the score received from our risk estimate model) exhibited a nine-fold increase in risk compared to women who scored in the bottom 20%.

**Table 2:** This table presents the results of testing the trained model on a test set of 2,015 women. Each woman in the test set was assigned a score representing the likelihood of that woman having breast cancer. The 2,015 women were then ranked according to the assigned score. This table presents statistics from the ranking, split into 5 quintiles. The result indicates that the top quintile is 9 times as likely to have an accurate prediction for breast cancer as the bottom quintile

Quintile	Number of Women with Breast Cancer	Number of Women without Breast Cancer	Total number of Women	Odds Ratio	95% confidence interval
5	310	93	403	3.47	(2.88,4.06)
4	221	182	403	1.45	(1.24, 1.67)
3	167	236	403	0.8	(0.74,0.87)
2	113	290	403	0.56	(0.49,0.62)
1	89	314	403	0.39	(0.36, 0.42)

**Fig. 4:**
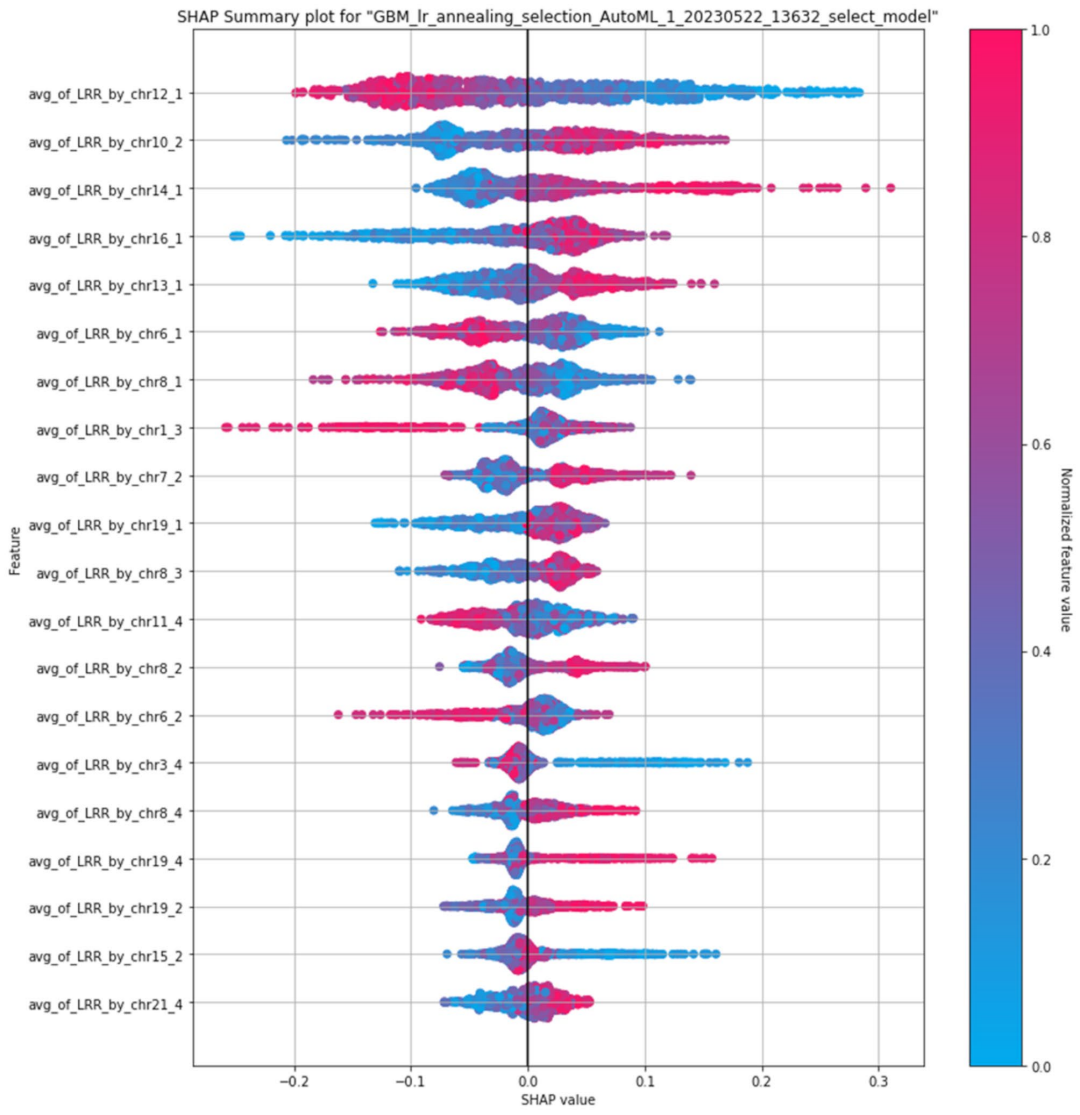
This Shapley additive explanations plot (known as a SHAP plot) provides interpretability to the machine learning model. In this model, we used the chromosomal-scale length variation on four segments from each chromosome, numbered from 1 to 4, for a total of 88 features. This plot shows the 20 most significant features. Each point represents an individual person. The color of the point indicates the normalized value, the red points (closer to 1.0) indicate the women with the “longest” associated chromosome-region, while the blue points (closer to 0) represent women with the shortest associated chromosome-region. The SHAP value indicates the relative importance of the datapoint to the prediction

To understand the model’s decision-making process and identify the chromosome regions that contributed most to these findings, we conducted a feature importance analysis using SHAP (Shapley Additive exPlanations). The SHAP process only works with certain types of models, and stacked ensemble models are not compatible. However, tree-based models do work with the SHAP process [33]. Among all the tree-based models, Gradient Boosting Model (GBM) ranked highest on the leaderboard and on a validation test. For that reason, SHAP analysis was conducted on a GBM to identify the influential regions. SHAP values were computed for all 88 CSLV features, and the 20 most important features are shown in Fig. 4. Each point represents an individual and is colored by the normalized chromosome segment length.

The All of Us dataset contains an element for “race,” which is self-identified by each person. Several hundred Black women in the dataset have been diagnosed with breast cancer, enabling us to characterize the difference between predictive models trained and applied to people of different self-identified races. This analysis allowed us to explore the transferability of the model across different self-identified racial groups and assess its generalizability.

We assessed the performance of the breast risk assessment model by training it on one self-identified racial group (White or Black) and testing it on different groups. Self-identified race distribution in both positive and control classes is displayed in Table 1. To conduct a cross-analysis considering all subpopulations in the positive class, we categorized the cases into three subgroups: “White”, “Black or African American”, and other. The “other” category includes cases with unknown self-identified race as well as individuals who self-identified as Asian, Middle Eastern, and Native Hawaiian groups. Due to the small number of cases in the Asian, Middle Eastern, and Native Hawaiian groups, they were not examined as distinct groups in the cross-analysis.

**Fig. 5:**
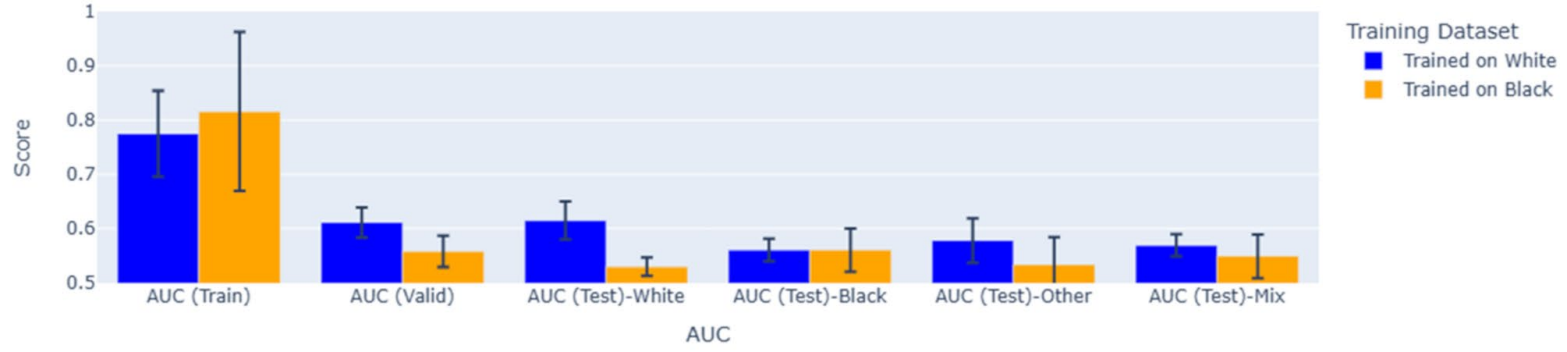
We trained two different machine learning models: one on a subset of women who self-identified as White and the other on an equal number of women who self-identified as Black. We recorded the AUC of these models during the training and validation of the models. We also tested these models on four different groups of women: who self-identified as (1) White, (2) Black, (3) a different category that was neither White nor Black and (4) a mixture of different categories. In all cases, we repeated the AUC measurements five times and mean AUCs along with the standard deviation is plotted

As we did with previous tests, we randomly selected an age-matched number of cases from the negative class within each subgroup for each run. Two machine learning models were developed in this study. The first model was trained on 80% of the finalized dataset from the White subgroup and evaluated on the remaining 20% split of the White population. Additionally, this trained model was evaluated on datasets of Black or African American individuals, “other” groups, and a combined dataset including White, Black, and “other” groups. Similarly, the second model was trained on 80% of the Black or African American subgroup and evaluated by testing its performance on the remaining 20% split of the Black subgroup, the White subgroup, “other” subgroups, and the combined dataset of White, Black, and “other”. Figure 5 compares the AUC of ROC plots for the two types of developed ML models during both training and validation steps.

## Discussion

In this study, we described a method of computing genetic risk scores for breast cancer as an alternative to traditional SNP-based algorithms. We used two approaches, first using one number computed per chromosome, then increasing to four numbers per chromosome. The first approach had an AUC of 0.60, comparable to current SNP-based genetic risk scores. When we increased the number of parameters computed per chromosome to four, we saw a significant increase in AUC to 0.70. Increasing the number of segments per chromosome significantly increases computational costs, processing time, and memory requirements, particularly given the size of the All of Us dataset. Using four segments per chromosome provided a reasonable balance, allowing us to capture additional structural variation beyond a single value per chromosome, while keeping the analysis feasible within available computational resources. While we recognize that averaging log R ratio (LRR) values across large chromosomal segments may mask fine-scale regional variations, our approach was intentionally designed to focus on large-scale structural variability rather than local polymorphisms. Averaging across millions of SNP markers mitigates the influence of random noise inherent in microarray measurements and reduces the impact of localized technical artifacts. By using chromosomal-scale segment averages, we aimed to capture stable, large-scale genomic patterns that may contribute to breast cancer risk but are typically missed by conventional SNP-based polygenic risk scores.

Importantly, this work serves as a proof-of-concept study demonstrating that structural variations, at a broader genomic scale, can meaningfully improve risk prediction in a large diverse dataset like All of Us. Although we did not apply additional masking or filtering strategies to exclude problematic genomic regions in this initial analysis, the aggregate nature of our method provides inherent robustness against small-scale technical artifacts. This suggests that we could further improve the AUC by combining it with a SNP-based score, increasing the training population, and potentially increasing the number of parameters we extract from the chromosomes in future studies with greater computational capacity. Additionally, more refined approaches, such as incorporating regional quality control filters, excluding problematic probes, or weighting chromosome segments based on probe density and measurement quality, could further enhance the predictive performance of CSLV methods.

To put our result in context, one of the most complete studies on breast cancer polygenic risk scores developed a prediction algorithm that had an AUC of 0.63 for women of European ancestry. This result was computed using a training set of about 94,000 positive cases and 75,000 controls [9]. In this work, we demonstrated an AUC of 0.70, with a training set about 5% of the size of that study. Given a baseline AUC of 0.50, our result represents about a 50% increase in predictive accuracy over that study.

Previously, we performed a similar analysis using the UK Biobank [24]. In that work, we found an AUC of 0.836 (95% CI 0.830, 0.843), which was due to a deep learning model. The best non-deep learning model (GBM) in that work had an AUC of 0.69, consistent with this work. Deep learning models are notoriously difficult to tune, and it is certainly possible that one could find a deep learning model using the All of Us data with a comparable AUC.

Our result for women of African ancestry was comparable to [9], we found an AUC of about 0.57, similar to [9]. In our work, we based this on only 440 positive cases of Black women with breast cancer, while in [9], they had substantially more. One recent paper [34] developed a risk prediction algorithm specifically for women of African ancestry. Using a dataset with 18,034 cases of female breast cancer in women with African ancestry (85.3% from U.S., 14.7% from the African continent and Barbados). They found a polygenic risk score with an AUC of 0.60 (95% CI 0.58–0.62).

Our machine learning model experiments based on ancestry (White or Black) yielded intriguing results. As depicted in Fig. 5, we trained machine learning models exclusively on either White or Black women. We then evaluated the effectiveness of these models on four distinct groups: solely White women, solely Black women, a group labeled “others” which included every woman in the dataset who classified herself as neither “Black” nor “White,” and a mixed group of women, which included every woman in the dataset.

Not surprisingly, we observed that models trained on White women performed significantly better on the White women compared to models trained on Black women. However, when these models trained on White women were tested on Black women, there was no significant difference in performance between models trained on White or Black women.

To further investigate the potential for model generalizability across different subgroups of races, it would be beneficial to include more data points from the Black subgroup. By increasing the sample size and diversity within the Black race, it is possible to enhance the model’s performance and explore its effectiveness in predicting breast cancer risk within this population.

The analysis of SHAP values and variable importance calculations indicates that there is no single chromosomal region that contributes significantly more than others to the predictions of the model. Instead, the model relies on a combination of multiple chromosomal regions and genetic factors to make accurate predictions. This suggests that the overall genomic landscape and interplay between various structural variations play a crucial role in determining the risk of breast cancer.

In our simulation of a real-world application of the model and its clinical use, women in the top 20% of the risk distribution based on our model had a nine-fold increased likelihood of breast cancer compared to those in the bottom 20%, suggesting that our approach could meaningfully stratify patients by risk. This level of stratification could help prioritize individuals for enhanced surveillance, earlier imaging, or preventive interventions.

In conclusion, we found that this method of computing genetic risk scores for breast cancer in the NIH All of Us dataset had an AUC of 0.70 (95% CI, 0.67–0.73) and women in the top quintile had an odds ratio about 9x higher than women in the lowest quintile. In addition, we compared models trained on populations of only White women and only Black women. We found that the models trained only on White women performed better than models trained only on Black women when tested on only White women, not surprisingly. Surprisingly, we did not see a significant difference between the two models when tested on only Black women.

## Data Availability

No datasets were generated or analysed during the current study.
